# Neuroprotective Effect of Zataria Multiflora Essential Oil on Rats With Alzheimer Disease: A Mechanistic Study

**DOI:** 10.32598/bcn.9.10.270

**Published:** 2019-01-01

**Authors:** Narges Eskandari-Roozbahani, Tahoora Shomali, Mahnaz Taherianfard

**Affiliations:** 1. Department of Basic Sciences, School of Veterinary Medicine, Shiraz University, Shiraz, Iran.

**Keywords:** Alzheimer Disease, Acetylcholinesterase, Brain-derived neurotrophic factor, Antioxidants

## Abstract

**Introduction:**

Finding herbs with promising effects to prevent or postpone Alzheimer Disease (AD) is highly demanded. The present study aimed at clarifying plausible effects and related mechanism(s) of Zataria Multiflora Essential Oil (ZMEO) against memory impairment in a rat model of the AD.

**Methods:**

Forty male adult rats were categorized into four groups and treated as follows: 1. The Negative Control (NC): no treatment; 2. Sham control (sham): distilled water by Intracerebroventricular (ICV) injection; 3. The AD control (AD): Aβ 1–42 by ICV injection; and 4. The ZMEO group: Aβ 1–42 by ICV injection and ZMEO at 100 μL/kg/d orally for 20 days.

**Results:**

After Congo red staining of the hippocampus, a relative decrease in amyloid deposits was observed in the ZMEO group. Moreover, rats showed better outcomes in Morris Water Maze (MWM) test, reduced hippocampal acetylcholinesterase (AchE) activity, and higher Brain-Derived Neurotrophic Factor (BDNF) content as compared with the AD group (P<0.05). However, no significant changes in antioxidant status was observed (P>0.05).

**Conclusion:**

ZMEO has a protective effect against memory impairment in rats with AD at least partly via reducing hippocampal AchE activity and enhancement of BDNF levels without a change in antioxidant status. These findings can pave the way for future studies on the usefulness of this herb in AD prevention.

## Highlights

- Zataria multiflora Essential Oil (ZMEO) has a protective effect against memory impairment in rats with Alzheimer disease.
- The effect is at least partly via reducing hippocampal acetylcholinesterase activity.
- Enhancement of brain-derived neurotrophic factor level can also be an underlying mechanism.
- Change in antioxidant status is not associated with ZMEO effects.

## Plain Language Summary

Finding herbs with promising effects to prevent or postpone Alzheimer Disease (AD) is highly demanded. The present study aimed at clarifying possible effects and related mechanism(s) of Zataria multiflora Essential Oil (ZMEO) against memory impairment in a rat model of the AD. Induction of AD was performed by injection of Aβ 1–42 in brain ventricles. Then the affected rats were treated with by oral administration of ZMEO for 20 days. At the end of the experiment, the treated rats showed better outcomes as shown by their less memory impairment. The effect of ZMEO was associated with reduced hippocampal acetylcholinesterase activity and higher brain-derived neurotrophic factor content. These findings can pave the way for future studies on the usefulness of this herb in AD prevention.

## 1. Introduction

Alzheimer Disease (AD) is a neurodegenerative condition and the leading cause of dementia. It was estimated that 46.8 million people worldwide were afflicted with dementia in 2015 and this number almost doubles every 20 years, reaching 74.7 million in 2030 and 131.5 million in 2050 due to global population aging (Prince et al., 2015).

Hippocampus, especially the CA1 region, is one of the most important brain structures that play a critical role in spatial learning and memory (Li et al., 2012) and also is the major part of the brain affected by AD (Fellgiebel & Yakushev, 2011). The disease is characterized by the accumulation of intracellular Neurofibrillary Tangles (NFT), senile plaques in extracellular space, and neuronal loss (Parihar & Hemnani, 2004). The most significant component of senile plaques is an insoluble fragment of Amyloid β protein (Aβ) formed by misprocessing of an Amyloid Precursor Peptide (APP). Direct injection of pre-aggregated Aβ 1–42 fragment is a non-transgenic in vivo model of AD, which is extensively used to study the molecular, morphological, and behavioral aspects of the disease (Zussy et al., 2011).

The precise underlying mechanism(s) of Aβ-induced neuronal cell death in AD is still unclear. Several reports suggest that a bunch of factors accelerate the rate of progression of AD, with oxidative stress as one of the most prominent culprits (Jeong et al., 2013). Neural growth factors such as Brain-Derived Neurotrophic Factor (BDNF) (Nagahara et al., 2009), acetylcholine neurotransmitter and Acetylcholinesterase (AchE) activity (Abe, Casamenti, Giovannelli, Scali, & Pepeu, 1994; Postu et al., 2017) are also important determinants.

Two classes of medications are currently approved by the United States Food and Drug Administration (FDA) to manage AD, including the AchE Inhibitors (AchEIs) such as tacrine, donepezil, rivastigmine, and galantamine as well as the noncompetitive N-Methyl-D-Aspartate (NMDA) receptor antagonist, memantine (Bassil & Grossberg, 2017). Unfortunately, long-term use of these drugs are costly and have considerable side effects. Moreover, duration of their efficacy is limited and above all, a definitive cure could not be achieved (Forsyth, Surmon, Morgan, & Wilcock, 1989; Muller, 2007; Cacabelos, 2007; Mimica & Presecki, 2009).

Much effort is now focused on finding herbs especially with antioxidant and AChE inhibitory properties that may have a promising effect in prevention or postpone of AD development. Zataria Multiflora Boiss. (ZM) is a thyme-like plant belonging to the Lamiaceae family that grows wildly only in Central and Southern Iran, Pakistan, and Afghanistan (Hosseinzadeh, Ramezani, & Salmani, 2000). Z. multiflora is not only a popular food condiment, but also famous for its diverse beneficial health effects, including antinociceptive, antimicrobial, spasmolytic, anti-inflammatory, and antioxidant properties (Sajed, Sahebkar, & Iranshahi, 2013).

Phytochemical analysis of ZM has revealed that the main constituents of the dry plant are carvacrol (61.3%) and thymol (25.1%) (Saleem, Nazli, Afza, Sami, & Ali, 2004). Both of these compounds have AchE inhibitory properties (Jukic, Politeo, Maksimovic, Milos, & Milos, 2007). Majlesi et al. reported amelioration of Aβ-induced cognitive deficits in rats by ZM essential oil using Morris Water Maze (MWM) test without strictly examining the possible mechanism(s) of the observed effect (Majlesi, Choopani, Kamalinejad, & Azizi, 2012). Along with the confirmation of the protective effect of ZM essential oil, the current study aimed at exploring some possible mechanisms of this effect by considering antioxidant, AChE inhibitory activity, as well as its effect on the BDNF content of hippocampus in rats with the Aβ-induced AD.

## 2. Methods

### 2.1. Phytochemical analysis of ZM essential oil

Hydro-distilled essential oil of ZM leaves was purchased from Barij Essence Pharmaceutical Co., Iran. Phytochemical analysis of the essential oil was performed by Gas Chromatography/Mass (GC/MS) spectrometric method as previously described by Shomali, Raeesi, & Eskandari-Roozbahani (2016). Briefly, the procedure was accomplished by Varian 3400-Varian Saturn II GC/MS system, Canada. A DB-5 column (30 m×0.25 mm×0.25 μm film thickness) was used with an inlet pressure of 240 kPa. The column temperature was set to 60–250°C with 3°C increases per minute. Injector port temperature was 260°C and carrier gas was helium with the linear velocity of 31.5 cm/s. Mass range was 40–340 U. Ionization voltage of mass spectrometer and ionization source temperature were 70 eV and 270°C, respectively.

### 2.2. Animals and study design

Forty male adults Sprague Dawley rats (weighing 250±30 g) were maintained in a temperature-controlled room (23°C), on a 12:12 hour light/dark cycle with free access to commercial laboratory rodent feed and tap water. After a week of adaptation, they were randomly allocated into four equal groups (n=10 in each) and treated as follows: 1. The Negative Control (NC) group that did not receive any treatment during the experiment; 2. Sham control (sham) group that received distilled water by Intracerebroventricular (ICV) injection into lateral ventricles and vehicle (5% tween 80, Merck, Germany) by oral gavage for 20 consecutive days; 3. The AD control (AD) group that received Aβ 1–42 (Sigma-Aldrich, USA) by ICV injection; and 4. The treatment (ZMEO) group that received Aβ 1–42 by ICV injection and on the next day, ZM essential oil at 100 μL/kg/d of pure essential oil in the vehicle by oral gavages for 20 consecutive days.

The dosage of ZM essential oil was according to a previous study protocol (Majlesi et al., 2012). It should be mentioned that in a pilot study, ZM essential oil was administered by Intraperitoneal (IP) injection, which resulted in inflammatory reactions at the injection site that adversely affected the performance outcome of animals in MWM task. Therefore, the oral garaging route for the original study was selected. Procedures adopted in the present study were in accordance with institutional ethical guidelines for use of animals in experiments that were compatible with European convention for the protection of vertebrate animals used for experimental and other scientific purposes.

### 2.3. Induction of animal model of AD

Aβ 1–42 was dissolved in sterile distilled water at a concentration of 5 μg/μL. To obtain the aggregated form, the peptide solution was placed in an incubator at 37°C for 5–7 days. Light microscopic observation demonstrated the precipitation of insoluble globular aggregates (Ramin, Azizi, Motamedi, Haghparast, & Khodagholi, 2011).

Rats were anesthetized by IP injection of ketamine (100 mg/kg) (Alfasan, Netherland) and xylazine (8 mg/kg) (Alfasan, Netherland) cocktail. Then, they were placed in a stereotaxic instrument (Stoleting, USA) with the incisor bar set 3.5 mm below the inter-aural line. Skull was drilled and stainless-steel cannula (23-gauge) were implanted bilaterally into lateral ventricle (AP=−0.5, LR=1.5 mm, D=4 mm) (Paxinos & Watson, 2010). Tetracycline 3% was used on the skull immediately after surgery. The cannula was secured with stainless steel glass screws and dental acrylic cement.

One week after surgery, 2 μL of aggregated Aβ1–42 (10 μg) or vehicle (distilled water) was bilaterally infused into the lateral ventricle through a gauge cannula connected via polyethylene tubing to a 5-μL Hamilton microsyringe. The infusion speed was 0.5 μL/min and the needle remained in place for an extra one minute to avoid backflow (Gholamipour-Badie, Naderi, Khodagholi, Shaerzadeh, & Motamedi, 2013; with little modifications).

### 2.4. Morris water maze test

To investigate reference or spatial learning and memory in all rats, MWM test was initiated on the day 21 as previously described by Majlesi et al. A digital camera mounted above the water maze to capture images per second and transmit them to a personal computer running smart software (Neurovision, Iran) to calculate the escape latency, distance traveled, and average swim speed for each trial.

### 2.6. Visible platform training

To test spatial learning, three initial acquisition visual-training days were used to familiarize each animal with the water maze. The rat was allowed a maximum of 90 seconds to jump on the escape platform. The animals were then allowed to spend 15 seconds on the platform and 120 seconds in the home cage before the commencement of the next trial. During these days, the platform, placed in the center of the Southeast (SE) quadrant, was elevated 1.5 cm above the surface of the water and a gray flag was put on the platform. The platform position remained stable over three days and acquisition of this task was assessed. Each animal participated in four trials per day for three consecutive days. A different starting position was used for each trial performed on the same day and the sequence of starting positions was changed from day to day.

### 2.5. Reference memory acquisition

On day 4, each animal was tested for reference memory using the submerged invisible platform with a visual cue placed in a fixed position (SE). Each animal participated in four trials per day for three consecutive days using a procedure similar to that used for the training task.

On day 7, a probe test was performed, in which the platform was removed and the animals were allowed to search for it for 60 seconds. The time spent in the target quadrant (i.e. where the platform was located during the acquisition training sessions) were computed.

### 2.7. Sampling of hippocampal tissue

After accomplishing behavioral tests, all rats were sacrificed by decapitation under deep anesthesia. From the appropriate coronal sections the hippocampus was excised bilaterally and divided for Congo red staining or kept in −80°C for biochemical evaluation.

### 2.8. Congo red staining

Congo red staining was used to identify amyloid deposits in the hippocampus (Wilcock, Gordon, & Morgan, 2006). Briefly, the rehydrated brain sections were incubated in alkaline saturated NaCl (20 minutes), stained in alkaline Congo red reagent (Sigma-Aldrich, USA) for 35–40 minutes; then rinsed by dipping eight times in 95% ethanol, incubated for 15 minutes in three xylenes dishes and then mounted. Under a light microscope, the amyloid deposits showed red to orange color.

### 2.8. Preparation of tissue homogenate

Tissue samples (the hippocampus) were homogenized in 0.1 M phosphate buffer, pH 7.5, followed by centrifugation at 14000 rpm at 4°C for 5 min. Clear supernatants were used for the assay.

### 2.9. Acetylcholinesterase activity assay

Acetylcholinesterase activity assay kit (Sigma-Aldrich, USA) was used based on an optimized version of Ellman’s method. Tissue total protein concentration was determined by Bradford protein assay. Activity was presented as unit per gram total of protein in hippocampal tissue.

### 2.10. Evaluation of hippocampal BDNF content

Hippocampal level of BDNF was measured using a rat Enzyme-Linked Immunosorbent Assay (ELISA) kit (Mybiosource, USA), according to the manufacturer’s instructions. The detection limit for BDNF was 31.2 pg/mL.

### 2.11. Antioxidant status assays

The extent of lipid peroxidation in the hippocampus was determined quantitatively by measuring Malondialdehyde (MDA) content using a colorimetric method. The kit was prepared by Zellbio (Germany) and assay was performed as described by the manufacturer. Total Antioxidant Capacity (TAC) was assayed by a colorimetric method based on Ferric Reducing the Ability of Plasma (FRAP). The kit was purchased from Zellbio (Germany).

### 2.12. Statistical analysis

The obtained data were expressed as Mean±Standard Deviation (SD). The data of the first and second 3 training days with visible and hidden platforms were analyzed by repeated measures ANOVA followed by Bonferroni method as post hoc. Other data were analyzed by one-way ANOVA followed by Tukey’s multiple comparison tests. In all cases, differences were considered statistically significant at P<0.05.

## 3. Results

### 3.1. Phytochemical composition of ZM essential oil

Different constituents of the essential oil are presented in Table 1. Thymol and carvacrol were the major constituents each comprising more than 30% of the essential oil, followed by p-cymene with 9.5% (Table 1).

**Table 1.** Phytochemical Analysis of ZMEO

**Number**	**Compound**	**(%)**	**Kovats Index**
1	α–Thujene	0.261	930
2	α–Pinene	3.275	940
3	Comphen	0.183	954
4	β–Pinene	0.437	980
5	3-Octanone	0.345	987
6	Myrcene	0.901	992
7	α–Terpinene	1.796	1020
8	p–Cymene	9.575	1026
9	Limonene	0.384	1032
10	1.8-Cineole	0.571	1035
11	γ–Terpinene	5.413	1064
12	Terpinolene	0.204	1088
13	Linalool	1.232	1100
14	Terpinene-4-ol	0.696	1178
15	α–Terpineol	0.761	1190
16	Methyl ether thymol	0.957	1237
17	Methyl ether carvacrol	1.393	1245
18	Thymol	34.670	1292
19	Carvacrol	32.070	1300
20	Thymol acetate	0.786	1355
21	Carvacrol acetate	0.991	1373
22	E–caryophyllene	1.840	1418
23	Aromadendrene	0.763	1430
24	Viridiflorene	0.493	1495
	Total	100	

ZEMO; Zataria multiflora essential oil.

### 3.2. Behavioral results

**Figure 1.**
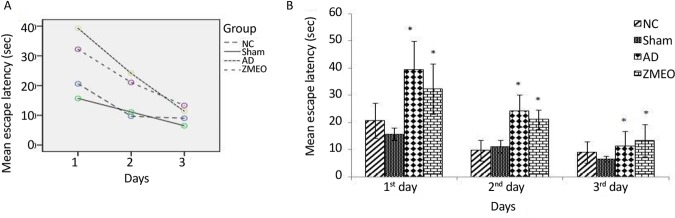
Mean escapes latency to find the visible platform in a water maze A) The learning patterns of the animals in different groups; B) The escape latency to the visible platform during days 1–3 of training; four groups were tested: The NC (negative control group), sham (sham control group), the AD (the AD control group), and the ZMEO (the ZM essential oil 100 μL/kg). Error bars indicate ±SD. * P<0.05 versus sham group.

**Figure 2.**
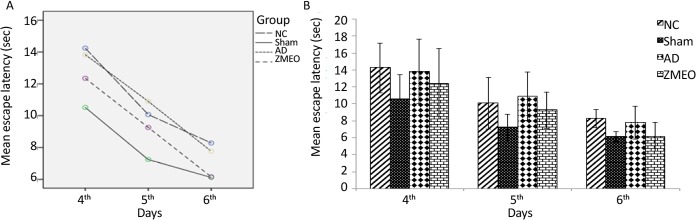
Mean escapes latency to find the invisible platform in a water maze A) The learning patterns of the animals in different groups; B) The escape latency to the invisible platform during days 4–6 of training; four groups were tested: the NC (negative control group), sham (sham control group), the AD (the AD control group), and the ZMEO (the ZM essential oil 100 μL/kg). No significant difference was observed among the groups. Error bars indicate±SD.

**Figure 3.**
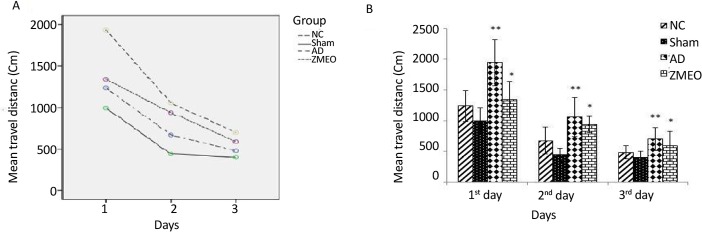
Mean travel distances to find the visible platform in a water maze A) The pattern of distance traveled by the animals; B) The travel distances to the visible platform during days 1–3 of training; the obtained data were averaged to reach a mean performance for each animal. Four groups were tested: the NC (negative control group), sham (sham control group), the AD (the AD control group), and the ZMEO (the ZM essential oil 100 μL/kg). Error bars indicate±SD. * P<0.02 versus the ZMEO and SC groups. ** P<0.0001 versus the AD and SC groups.

**Figure 4.**
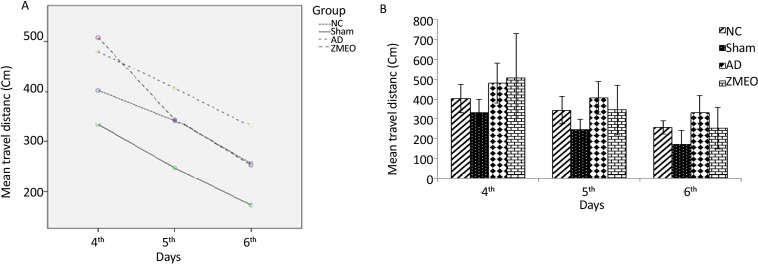
Mean travel distances to find the invisible platform in a water maze A) The pattern of distance traveled by the animals; B) The travel distance to the invisible platform during days 4–6 of training; the obtained data were averaged to reach a mean performance for each animal; four groups were tested: the NC (negative control group); sham (sham control group), the AD (the AD control group), and the ZMEO (the ZM essential oil 100 μL/kg). Error bars indicate±SD.

**Figure 5.**
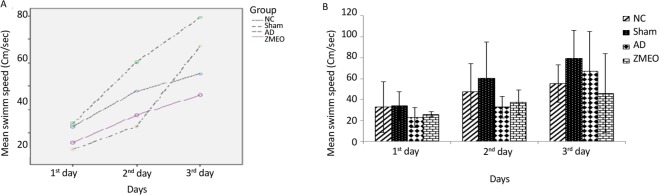
Mean swimming speeds to find the visible platform in a water maze A) The pattern of swimming speed by the animals; B) The swimming speed to find the visible platform during days 1–3 of training; there was no significant difference among groups. The data were averaged to obtain a mean performance for each animal. Four groups were tested: the NC (negative control group), sham (sham control group), the AD (the AD control group), and the ZMEO (the ZM essential oil 100 μL/kg). Error bars indicate±SD.

**Figure 6.**
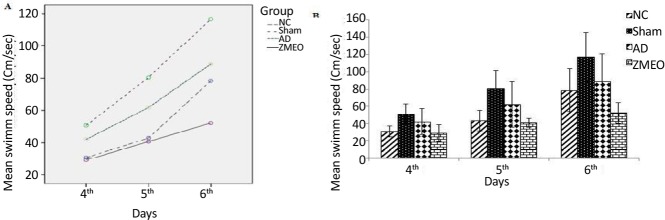
Mean swimming speeds to find the invisible platform in a water maze A) The pattern of swimming speed by the animals; B) The swimming speed to find the hidden platform during days 4–6 of training; there was no significant difference among groups. The data were averaged to obtain a mean performance for each animal. Four groups were tested: the NC (negative control group), sham (sham control group), the AD (the AD control group), and the ZMEO (the ZM essential oil 100 μL/kg). Error bars indicate±SD.

There was a negative linear correlation between escape latency (Figures 1 and 2A) and travel distance (Figures 3 and 4A) with training days in all groups as shown by significant differences in the day 2/day 1 and the day 3/day 2 values (P<0.001 for all comparisons), indicating that all groups in invisible and hidden platforms training learnt the platform location. Moreover, a positive correlation was observed between swimming speed and training days with significant differences between two consecutive days (P<0.001) (Figures 5 and 6A).

### 3.3. Escape latencies invisible platform training

There was a significant difference in escape latencies among groups during three days of training with the visible platform (F_4,24_=4.325, P=0.009). According to further analysis using post hoc test, no significant difference was observed between the NC and sham groups in three testing days (P>0.05), while there was a significant increase in escape latency of rats in the AD and the ZMEO groups in first and second days as compared with the sham group (P<0.001). Rats in the ZMEO group showed increased escape latency as compared with sham group in the third day (P=0.002). No significant difference was observed between the AD and the ZMEO groups in three testing days (P>0.05) (Figure 1 B).

### 3.4. Escape latencies in hidden platform training (reference spatial memory)

As shown in Figure 2 B, there was no significant difference in escape latencies among the groups during the three testing days (F_6,38_=0.706, P=0.647).

### 3.5. Travel distance in visible platform training

Repeated-measures analysis showed significant differences in travel distances during three testing days among the groups (F_4,23_=2.95, P=0.041). More analysis using Bonferroni method revealed a significant increase in distance traveled by the AD and the ZMEO groups during this test as compared with sham (P<0.001 and <0.02, respectively) and the AD compared with the NC group (P=0.002) at three testing days. No significant difference was observed between the AD and the ZMEO groups (P>0.05) (Figure 3 B).

### 3.6. Travel distance in hidden platform training (reference spatial memory)

During the reference spatial memory test with the invisible platform, there was no significant difference in travel distance among the groups (F_6,34_=1.392, P=0.246) (Figure 4 B).

### 3.7. Swimming speed in visible and hidden platform training

As shown in Figures 5 B and 6 B, there was no significant difference in swimming speed among groups during the days 1–3 (F_6, 60_=0.911, P=0.494) and the days 4–6 (F_4,31_=1.845, P=0.139).

### 3.8. Probe test

**Figure 7.**
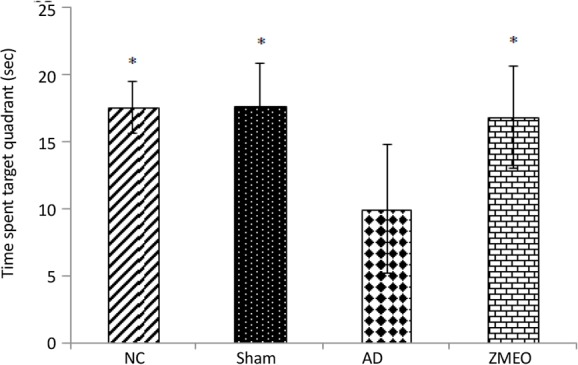
Mean time spent in target quadrant of water maze in the day 7 (24 hours after learning trails) The data were averaged to obtain a mean performance for each animal. Four groups were tested: the NC (negative control group), sham (sham control group), the AD (the AD control group), and the ZMEO (the ZM essential oil 100 μL/kg). Error bars indicate±SD. * P<0.05 versus the AD group.

In this test, the platform was removed and time spent in target quadrant of MWM was recorded. One-way ANOVA showed a significant difference in time spent in the target quadrant of MWM among the groups in day 7. Further analysis using Tukey post hoc test revealed a significant decrease in time spent in the target quadrant of the AD group as compared with the NC) P=0.007) and sham (P=0.012) groups. Rats in the ZMEO group spent significantly longer time in target as compared with the AD group (P=0.019). Interestingly, rats in the ZMEO group had statistically the same result as those were in the NC or sham groups (P>0.05) (Figure 7).

### 3.9. Histological results

**Figure 8.**
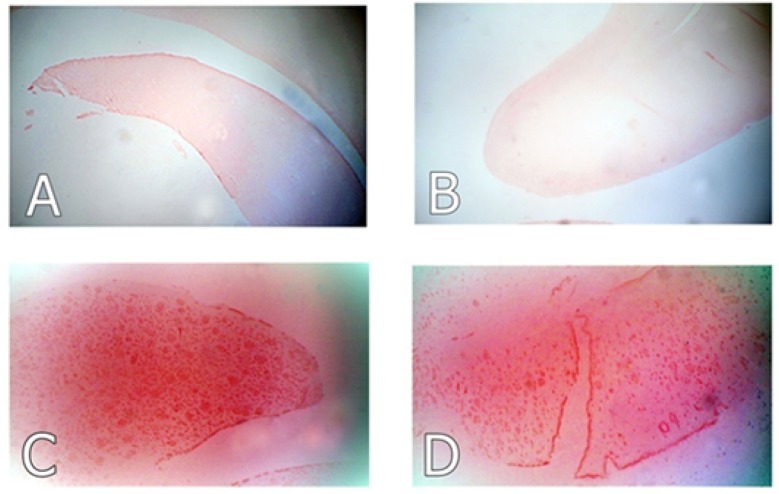
Photo micrographs of Congo red stained sections of hippocampus in different groups Representative photo micrographs of Congo red stained sections of hippocampus in (A) the negative control, (B) sham control (C) the AD control (AD, Aβ 1–42 by ICV injection) and (D) the treatment group (Aβ 1–42 I.C.V injection plus ZM essential oil by oral gavages). High density of amyloid deposits (orange-red plaques) was observed in the AD group. The severity of plaque deposition was subjectively lower in the treatment group. No plaques were detected in the negative and sham control groups (Magnification: 100×).

As shown in Figure 8, rats in the AD and the ZMEO groups that received Aβ 1–42 by injection into lateral ventricles, showed orange-red amyloid plaques, which were subjectively seemed more pronounced in the AD group, although no quantitative evaluation on the number or size of plaques were performed. The hippocampal tissue of the NC and sham groups was void of any plaque deposits.

### 3.10. Activity of AchE

**Figure 9.**
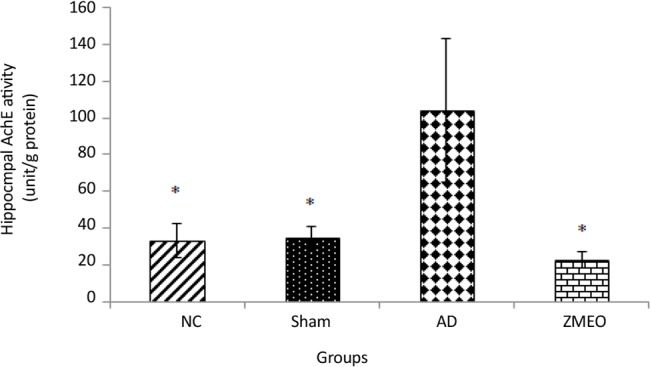
Mean AchE activity in hippocampus from rats in different groups four groups were tested: the NC (negative control group), sham (sham control group), the AD (the AD control group), and the ZMEO (the ZM essential oil 100 μL/kg). Error bars indicate±SD. *P<0.001 versus the AD group.

Figure 9 shows that the enzyme activity significantly increased in the AD group as compared with the NC or sham groups (P<0.001). Rats in the ZMEO group showed a significantly decreased activity of the enzyme in hippocampal tissue as compared with the AD group (P<0.001). Interestingly, no significant difference was observed between the ZMEO, and NC or sham groups (P>0.05).

### 3.11. BDNF concentration

**Figure 10.**
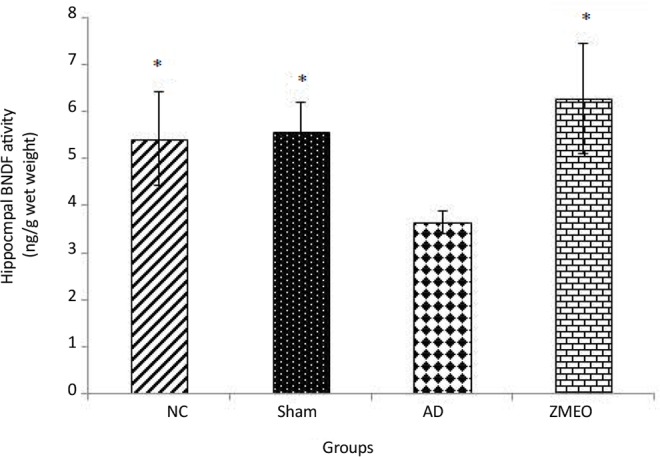
Mean BDNF concentration (ng/g w/w) in hippocampus homogenate from rats in different groups Four groups were tested: the NC (negative control group), sham (sham control group), the AD (the AD control group), and the ZMEO (the ZM essential oil 100 μL/kg). Error bars indicate±SD. * P<0.05 versus the AD group.

The BDNF concentration significantly decreased in the AD group as compared with the NC (P<0.02) or sham (P<0.01) groups (Figure 10). Rats in the ZMEO group showed a significantly increased content of BDNF in hippocampal tissue as compared with the AD group (P<0.001). Interestingly, no significant difference was observed between the ZMEO, and the NC or sham groups (P>0.05).

### 3.12. Antioxidant status

**Figure 11.**
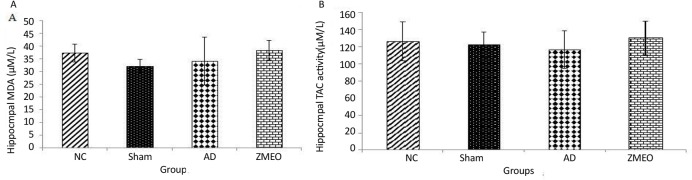
Mean MDA content (A) and TAC (B) in hippocampus homogenate from rats in different groups There was no significant difference among groups (P>0.05). Four groups were tested: the NC (negative control group), sham (sham control group), the AD (the AD control group), and the ZMEO (the ZMEO 100 μL/kg). Error bars indicate ±SD.

Figure 11 (A and B) shows no significant difference in the oxidative stress markers among groups (P>0.05).

## 4. Discussion

In the current study, some probable mechanisms behind the protective effect of ZM essential oil in the AD with regard to certain factors playing a role in the evolution and progression of the disease were assessed. An appreciable spatial memory impairment was observed after 20 days following ICV injection of Aβ1–42 (10 μg in 2 μL) into lateral ventricles of the rat’s brain as shown by results of the probe tests. The validity of this model was also supported by Congo red staining, revealing amyloid deposition in the hippocampus of the AD rats.

As previously stated, Majlesi et al. for the first time described ameliorative effects of ZM essential oil on Aβ-induced cognitive deficits in rats using MWM test without examining the possible underlying mechanisms (Majlesi et al., 2012). The current study clearly confirmed this report where a relative decrease was observed in amyloid deposits in the ZMEO group as compared with the AD group and a better outcome of the rats treated with ZM essential oil, in MWM test. Moreover, the present study demonstrated that ZM essential oil treatment partially restores spatial memory function through its ability to reduce hippocampal AchE activity accompanied by restoration of BDNF content to normal levels.

Regarding the swimming speed, which is used as a principle for locomotor activity, the present study results were consistent with the previous studies (Ramin et al., 2011; Majlesi et al., 2012). It was shown that learning impairments in the MWM test were independent of locomotor effects, because land-based locomotor deteriorations do not affect swimming speed. Moreover, when the experimental animals have deficits during probe trials, this further dissociates learning from performance, because measures recorded on probe trials are insensitive to swimming speed (Fitzgerald & Dokla, 1989). In the assessment of reference spatial memory, there was no significant difference in escape latency and traveled distances among groups. These findings suggest that reference spatial memory was not impaired by Aβ injection in the lateral ventricles and are in accordance with the report by Gholamipour-Badie et al.

It was well established that Aβ injection into rat brain impairs learning as a result of a hypofunction of the hippocampal cholinergic system caused by a reduction in acetylcholine release mechanisms (Abe et al., 1994). In a closely related recent study, Postu et al. observed a significant increase in hippocampal AchE activity of rats after about three weeks of ICV injection of Aβ 1–42, which is in close agreement with the current study findings in the AD group. The results obtained from GC/MS analysis in the present study were consistent with previous research findings (Saleem et al., 2004). Carvacrol and thymol both show the AchEI effect; the activity of carvacrol is more than thymol (Jukic et al., 2007).

Acetylcholinesterase inhibitors are the mainstay of current pharmacotherapy for the AD (Lieo, Greenberg, & Growdon, 2006). Since current therapeutic strategy in the AD was to restore cholinergic function through inhibition of AchE and thereby facilitating cholinergic neurotransmission, it is likely that AchEI activity of carvacrol and thymol, as the most important constituents of ZM essential oil, are contributed to its beneficial effects. Thus, the protective effect of ZM essential oil on spatial memory function may be related to its ability to counteract the harmful changes in levels of critical cholinergic neurotransmitter-induced by Aβ peptide.

In the present study, Aβ could reduce BDNF level in the hippocampus. The effect of Aβ 1–42 on hippocampal levels of BDNF was controversial and might be related to the site of Aβ 1–42 injection and or time of post-injection sampling. For instance, in Christensen et al. study, rats injected bilaterally into the dorsal hippocampus with Aβ 1–42 showed a decrease in BDNF of frontal cortex without a significant change in hippocampal levels as compared with control rats after about 80 days post-injection (Christensen, Marcussen, Wörtwein, Knudsen, & Aznar, 2008). On the contrary, Postu et al. reported a significant decrease in hippocampal mRNA copy numbers of BDNF after about three weeks of ICV injection of Aβ 1–42 (Postu et al., 2017).

Previous studies provide support for neurotrophic factors as promising drug targets of the AD by proving that BDNF can prevent the death of injured adult neurons in the hippocampal formation, cortex, and basal forebrain (Murer, Yan, & Raisman-Vozari, 2001). According to this view, decreased levels of BDNF could contribute to the neurite atrophy and synaptic loss observed in the brains of patients with AD (Nagahara et al., 2009). Levels of BDNF could be enhanced by dietary manipulations such as calorie restriction or consumption of antioxidant diet (Fahnestock et al., 2012). It was observed in the present study that hippocampal BDNF levels of rats treated with ZM essential oil were appreciably higher than those of the AD group. Therefore, enhancement of BDNF content may play a role in the better outcome of rats treated with ZM essential oil in performing MWM task.

Oxidative stress plays an important role in the development and progression of the AD (Reddy, 2006). Aβ interacts directly with the mitochondria and induces production of free radicals, mitochondrial dysfunction, and cell death (Reddy, 2006). Thus, antioxidants may have a protective effect against learning and memory deficits induced by Aβ. However, the results of Aβ oxidative effects are contradictory in various studies (Cetin & Dincer, 2007; Miranda, de Bruin, Vale, & Viana, 2000).

One of the plausible reasons is that only one selective antioxidant may change during the AD; for example, it is reported that vitamin E is the most common antioxidant changes in the AD. In the present study, no appreciable change was found in the hippocampal antioxidant status of rats in the AD and ZM essential oil did not show a positive effect on MDA or TAC levels, suggesting that the beneficial effect of ZM essential oil on learning and memory does not depend on its antioxidant capacity.

In conclusion, it seems that the protective effect of orally administered ZM essential oil on learning and memory impairment due to ICV injection of Aβ 1–42 in rats, as shown by MWM task outcome and Congo red staining, is at least partly via reducing hippocampal AchE activity accompanied by enhancement of BDNF levels without a change in antioxidant status.

## Ethical Considerations

### Compliance with ethical guidelines

Procedures adopted in the present study were in accordance with institutional ethical guidelines for use of animals in experiments that were compatible with European convention for the protection of vertebrate animals used for experimental and other scientific purposes.
